# Improving Hydrolytic Activity and Enantioselectivity of Epoxide Hydrolase from *Phanerochaete chrysosporium* by Directed Evolution

**DOI:** 10.3390/molecules29204864

**Published:** 2024-10-14

**Authors:** Huanhuan Shao, Pan Xu, Xiang Tao, Xinyi He, Chunyan Pu, Shaorong Liang, Yingxin Shi, Xiaoyan Wang, Hong Feng, Bin Yong

**Affiliations:** 1 College of Life Sciences, Sichuan Normal University, Chenglong Avenue, Chengdu 610101, China; wsshforget@sicnu.edu.cn (H.S.); panxu1992@126.com (P.X.); taoxiang@sicun.edu.cn (X.T.); hexy@sicnu.edu.cn (X.H.); tkaishui@163.com (C.P.); lrong2555@163.com (S.L.); sssiyx@163.com (Y.S.); wangxiaoyan0317@163.com (X.W.); 2 Sichuan Institute of Atomic Energy, Yidu West Road, Chengdu 610101, China; 3 College of Life Sciences, Sichuan University, Wangjiang Road, Chengdu 610199, China; hfeng@scu.edu.cn

**Keywords:** *Phanerochaete chrysosporium*, directed evolution, epoxide hydrolases, enantioselectivity, molecular docking

## Abstract

Epoxide hydrolases (EHs) catalyze the conversion of epoxides into vicinal diols. The epoxide hydrolase gene from *P. chrysosporium* was previously cloned and subjected to site-directed mutation to study its enzyme activity, but the results were unsatisfactory. This study used error prone PCR and DNA shuffling to construct a PchEHA mutation library. We performed mutation-site combinations on PchEHA based on enzyme activity measurement results combined with directed evolution technology. More than 15,000 mutants were randomly selected for the preliminary screening of PchEHA enzyme activity alongside 38 mutant strains with increased enzyme activity or enantioselectivity. Protein expression and purification were conducted to determine the hydrolytic activity of PchEHA, and three mutants increased their activity by more than 95% compared with that of the wt. After multiple rounds of screening and site-specific mutagenesis, we found that F3 offers the best enzyme activity and enantioselectivity; furthermore, the molecular docking results confirmed this result. Overall, this study uncovered novel mutants with potential value as industrial biocatalysts.

## 1. Introduction

An epoxide hydrolase (EH, EC3.3.2.3) is a type of enzyme that catalyzes the hydrolysis of epoxides into corresponding vicinal diols [1]. EHs exhibit high enantioselectivity and are capable of achieving the hydrolytic kinetic resolution of racemic epoxides, resulting in the production of optically pure diols and epoxides. These optically pure compounds are valuable intermediates for the synthesis of drugs with enhanced efficacy and reduced side effects [2,3]. Chiral epoxides and vicinal diols play an important role in the synthesis of optically pure drugs, and the distinct stereochemical configurations of these epoxides result in different applications and functionalities. For example, (R)-2-methyl glycidyl toluene sulfonate is always used as a chiral structural unit to prepare a TAK-281 drug for the treatment of traumatic and ischemic central nervous system injuries [4], whereas (S)-glycidyl toluene sulfonic acid can synthesize active oral HIV-1 protease inhibitors [5]. (R)-epichlorohydrin serves as a key synthesized chiral intermediate of (S)-metonol, apnor, and other drugs [6], and (S)-epoxychloropropane is the raw material of atorvastatin, the most widely used lipid-lowering clinical drug [7].

**Figure 1:**
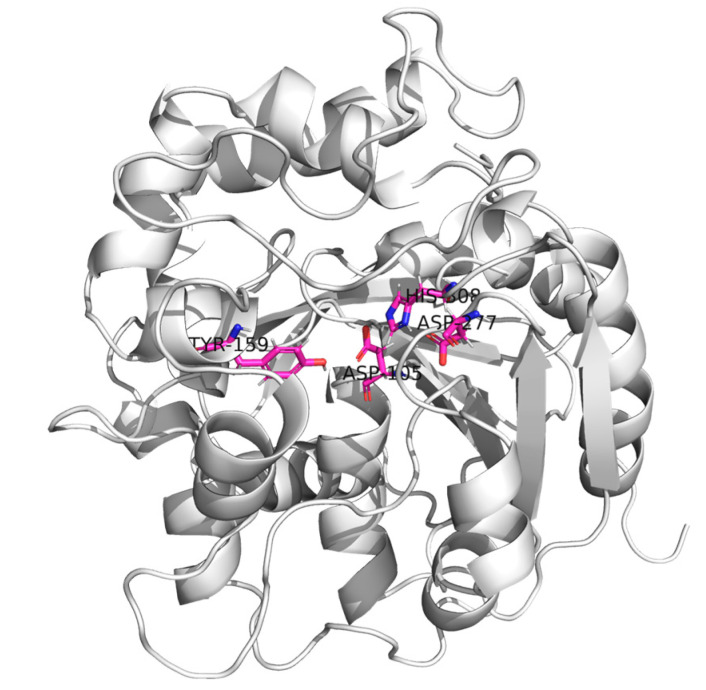
Schematic view of the 3D structure of the epoxide hydrolase from *Phanerochaete chrysosporium*. α-helices, β-strands, and coils are represented by helical ribbons, arrows, and ropes, respectively. The catalytic triad (D105 H308 D277) and tyrosine residue (Y159) are shown by sticks.

Enhancing the enzyme activity and enantioselectivity of EHs is a crucial approach for achieving higher yields of optically pure drugs. Most reported EHs are α/β folding hydrolases [8,9], whose structural framework is fundamentally conserved (Figure 1). These EHs include a core domain characterized by β-sheets surrounded by α-helices and a variable cap domain positioned above the core domain [10]. The active center of EHs is located at the junction of the main domain and cap domain, including the conservative catalytic ternary Asp-His-Asp/Glu [11,12]. The active sites of EHs also include two tyrosines located in the cap field; these tyrosines act as proton donors of epoxide atoms and assist in the ring opening of the epoxide [13]. Directed evolution is a powerful tool for inducing gene mutations, primarily utilizing error-prone PCR to introduce mutations, DNase I cleavage for fragment generation, and assembly and reamplification by PCR to improve enzymes or proteins to fit our interests. Therefore, employing directed evolution to induce gene mutations, followed by protein structure analysis of EHs and site-directed mutagenesis, may hold significant potential for enhancing the enzymatic activity of EHs.

Therefore, during its secondary metabolic stage, *Phanerochaete chrysosporium* can express a series of extracellular oxidases that degrade lignin and various aromatic pollutants. Consequently, *P. chrysosporium* holds significant potential for applications in the paper industry and the biological treatment of environmental pollution [14]. Twenty genes encoding epoxide hydrolases have been annotated in the genomic sequence of *P. chrysosporium* RP78, representing the largest number among known species. This result indicates that *P. chrysosporium* may have a unique epoxide metabolism pathway or ability, simultaneously providing abundant gene resources for the study of epoxide hydrolases [15]. The epoxide hydrolase gene (*PchEHA*, GenBank ID: EU348855) from the white-rot basidiomycetes *P. chrysosporium* was previously cloned and mutated in a site-directed manner, but the improvement in its enzyme activity was relatively limited [12]. In this study, the *PchEHA* gene of *P. chrysosporium* was randomly mutagenized, and high-throughput screening of the constructed mutant library was performed. Mutants with significantly improved enzymatic activity were obtained via the preliminary screening and rescreening of enzyme activities. Site-directed mutagenesis and protein purification were used to characterize the function of the mutant. The results of this study will contribute to a better understanding of the catalytic mechanism of epoxides for PchEHA.

## 2. Results

### 2.1. Construction of the Mutation Library

**Figure 2:**
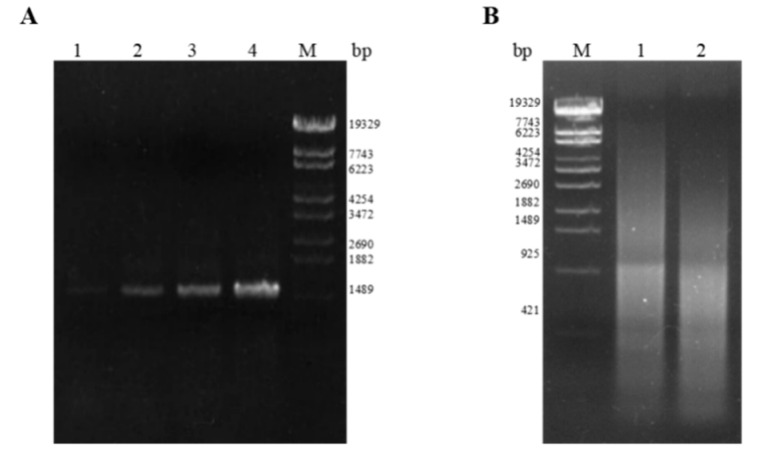
The *PchEHA* gene mutation library constructed via random mutation. (**A**) Electrophoresis analysis of mutant PCR products; dNTP/dITP in A1 lane is 25/175, dNTP/dITP in A2 lane is 50/150, dNTP/dITP in A3 lane is 75/125, and dNTP/dITP in A4 lane is 200/0; (**B**) electrophoresis analysis of PCR products without primer assembly, and DNA fragments obtained by EndoV enzymatic digestion serve as mutual primers in assembly PCR to enhance the mutational diversity of the DNA fragments. M: λ-EcoT14 I digest DNA Marker.

Random mutation of the wild-type (wt) epoxide hydrolase *PchEHA* gene was performed using error-prone PCR and DNA shuffling. The *PchEHA* gene mutation library (Figure 2) contained no fewer than 100,000 independent clones and exhibited a mutation rate of 100% based on sequencing, with an average of 5.09 base mutations per 1 kb and an average of 5.13 base mutations per individual PchEHA gene clone among 34 randomly selected colonies. All 34 clones were found to have a total of 174 mutation sites, with one clone containing 10 mutation sites—the largest number of sites among the samples. Statistics revealed that most mutations were conversions, accounting for 88.37% of the total mutation frequency, whereas transversion mutations accounted for only 11.63% (Table 1). The results showed that the mutation method adopted in this experiment could effectively cause random mutations of the *PchEHA* gene.

### 2.2. Screening of the Mutation Library 

More than 15,000 mutants were randomly selected for preliminary screening of their PchEHA enzyme activity, with 230 mutant strains exhibiting an increase in enzyme activity of over 20%, and 3 strains presenting enzyme activity exceeding 80% compared to the wild-type strain. These 230 strains were cultured in triangular flasks and used for re-screening, but only 66 strains had higher enzyme activity than that of the wild-type strain. In addition, the enzyme activity of 14 strains increased by between 20% and 50%. These 66 mutants of the *PchEHA* gene were then sequenced. The results showed that 38 mutants exclusively underwent amino acid site changes. 

**Figure 3:**
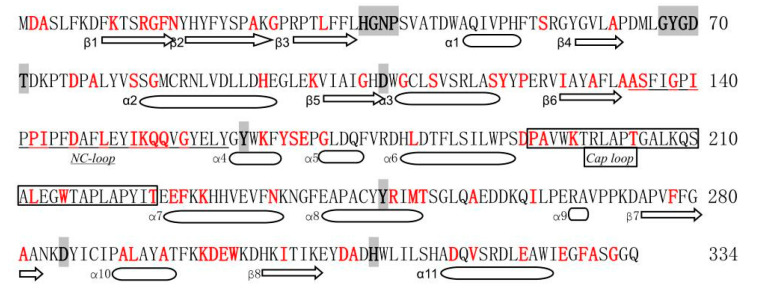
Labeling of the secondary structure of PchEHA amino acid and the mutation sites. The red font represents mutation sites, and the black background represents conserved sequences. 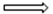 denotes β-sheets, 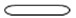 represents an α-helix.

A total of ninety-four mutation sites were present among these 38 mutants, and 25 mutants possessed only one or two amino acid mutation sites. However, 13 mutants had more than two mutation sites. Notably, mutant M37 exhibited the highest number of amino acid mutation sites, with a total of seven mutations (Table 2). Through a statistical analysis of amino acid positions, we observed the presence of the A189T mutation in three independent mutant strains, while A3V, S131P, K192M, A273T, and D305G were observed in two mutant strains. Other mutation sites were found to occur in only one mutant strain. Among the 94 mutation sites, alanine (M1, M6, M21) at position 189, lysine (M12, M22, M36) at position 192, and alanine (M2, M18, M24) at position 330 were all present in three mutants. In addition, eight positions were present in two mutants, indicating that these positions might have an important effect on the enzyme activity of PchEHA (Figure 3) [12]. 

### 2.3. Protein Expression of Mutants 

**Figure 4:**
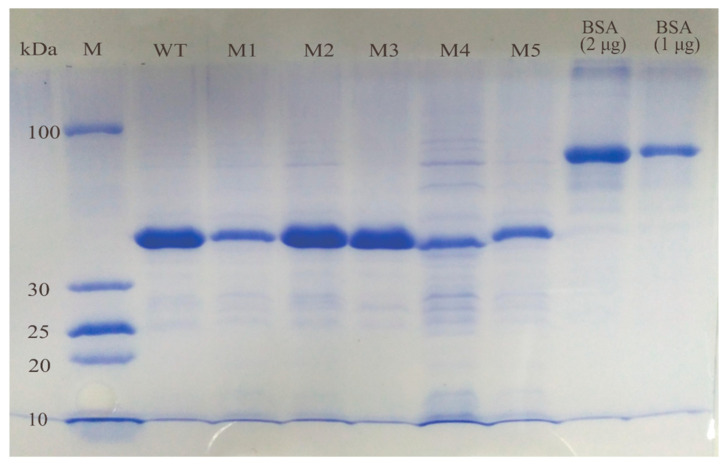
SDS-PAGE of purified wt PchEHA and 5 mutants. M: PageRuler^TM^ Protein Ladder (3.4–100 kDa); lane 2 is wt, lane 3–7 is M1–M5, lane 8 is protein bovine serum albumin (BSA) at a concentration of 2 μg, lane 9 is BSA at a concentration of 1 μg.

Based on the re-screened enzyme activity results and mutation site analysis, 32 mutants (M1–M32) were ultimately selected for protein expression and purification. The WT and 32 *PchEHA* mutant genes were well-expressed in *E. coli* BL21 (DE3) cells under the conditions described in the Materials and Methods section. Next, 12.5% SDS-PAGE electrophoresis was used to detect the purified proteins, with a clear band appearing between 30 kDa and 100 kDa, which was consistent with the size of PchEHA (41.3 kDa) (shown in Figure 4). 

### 2.4. Enzyme Activity Determination

**Figure 5:**
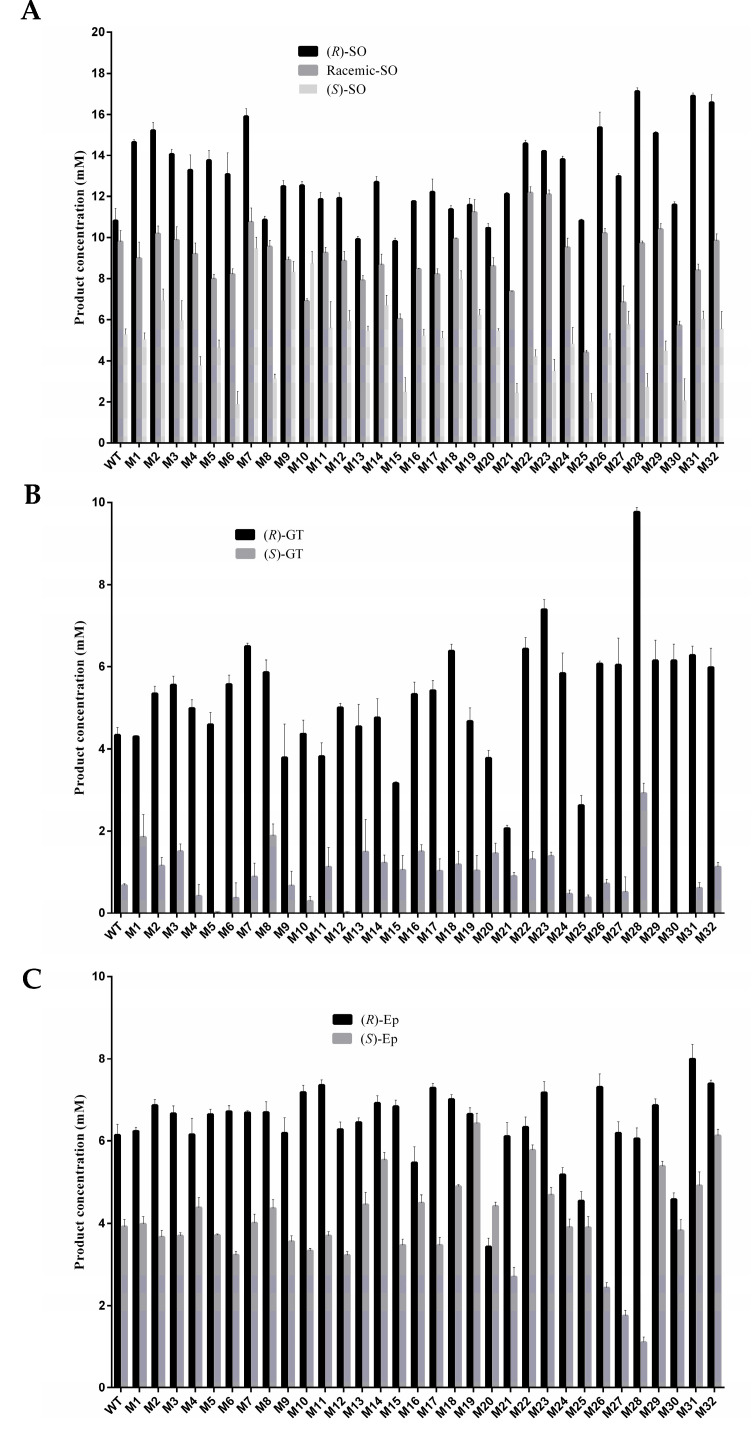
Hydrolytic activity of PchEHA wt and mutants to substrates. (**A**) The hydrolytic reactions were carried out in reaction volume containing (RS)/(R)/(S)-SO and purified enzymes; (**B**) the hydrolytic reactions were carried out in reaction volumes containing (R)/(S)-GT and purified enzymes; (**C**) the hydrolytic reactions were carried out in reaction volumes containing (R)/(S)-Ep and purified enzymes.

To determine the hydrolytic activity of PchEHA, six racemic epoxides, including (R)/(S)-Styrene oxide [(R)/(S)-SO], (R)/(S)-Epichlorohydrin [(R)/(S)-Ep] and (R)/(S)-Glycidyl tosylate [(R)/(S)-GT], were chosen as substrates (Figure 5). The hydrolysis rate of WT to (R)-SO and (S)-SO was 59.18% and 24.33%, respectively. Compared with the WT, 30 mutants presented increases in their (R)-SO hydrolytic activity. Among them, the hydrolysis rate of (R)-SO for three mutants (M28, M31, M32) increased by more than 95% compared with the wt. At the same time, the (S)-SO hydrolysis rate of 19 mutants improved, and four strains (M5, M9, M12, M30) experienced an increased rate exceeding 40%. Most of the mutants preferred (R)-SO to (S)-SO, and M6 experienced the highest increase in enantioselectivity (3.3 times). 

Nine mutant strains experienced a more than two-fold increase in their hydrolysis rates for (S)-GT compared with the wt, and only two mutant strains (M23 and M28) presented an increase in their hydrolysis rates for (R)-GT exceeding 70%. However, the hydrolysis rates for (R)-GT remained significantly higher than those for (S)-GT across all mutant strains. Unlike (R)/(S)-SO and (R)/(S)-GT, the hydrolysis rates of mutants on (R)/(S)-Ep did not significantly differ; for example, the hydrolysis rates of M32 on (R)-Ep and (S)-Ep were almost similar. This phenomenon also occurred in M29 and M14. 

### 2.5. Acquisition of the Combined Mutants and Determination of Protease Activity

M2, M25, M28, M30, M31, and M32 were selected as the initial mutants according to the above data on enzyme activity and the positions of the mutation sites among the screened mutants. Combination mutants were generated utilizing the mutation sites from these six mutant strains. Primers CS, YH, and EG were designed to carry out site-directed mutagenesis PCR to acquire 13 combination mutants. The target proteins were obtained via IPTG induction at 18 °C and purified using a nickel column ÄKTAPrime system. 

**Figure 6:**
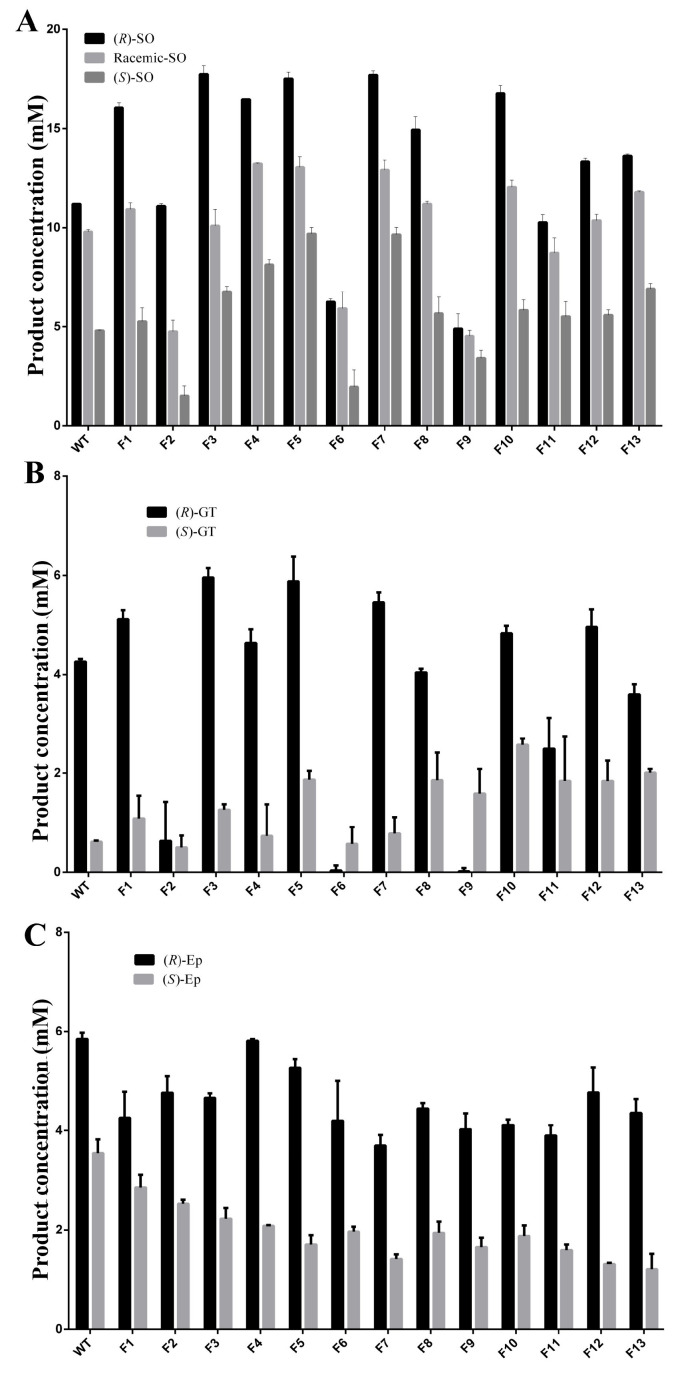
Hydrolytic activity of PchEHA wt and 13 combined mutants to substrates. (**A**) The hydrolytic reactions were carried out in reaction volumes containing (RS)/(R)/(S)-SO and purified enzymes; (**B**) the hydrolytic reactions were carried out in reaction volumes containing (R)/(S)-GT and purified enzymes; (**C**) the hydrolytic reactions were carried out in reaction volumes containing (R)/(S)-Ep and purified enzymes.

To determine the impact of recombination with different mutation sites on hydrolytic activity, racemic (R)- and (S)-SO, and (R)- and (S)-GT, were chosen as substrates. The results of the hydrolytic activity measurements showed that the hydrolysis rate of combination mutants F3, F5, and F7 to (R)-SO was greater than 90%. In addition, the hydrolytic activity of these mutants increased by more than 55% compared with that of the wt. When (S)-SO was used as the substrate, the hydrolytic activity of the F5 and F7 combination mutants increased to double the WT activity (Figure 6A). With the (S)-GT substrate, WT preferentially hydrolyzed (R)-GT. The combination mutants F5, F8, F10, F11, F12, and F13 experienced 2–3-fold hydrolytic activity increases in (S)-GT. However, the enantioselectivity of GT was reduced and (S)-GT was preferentially hydrolyzed for the combination mutants F6 and F9. The hydrolysis activity of F9 to (S)-GT was 32.84 times greater than that of (R)-GT (Figure 6B). When (R)- was replaced with (S)-Ep as the substrate, the activity of 13 mutants decreased compared with that of the wt. Although the hydrolytic activity of the F5, F12, and F13 combination mutants decreased, the hydrolytic activity against (R)-EP was more than three times that of (S)-Ep. For all combination mutants, enantioselectivity improved compared with that of the wt (Figure 6C).

### 2.6. Kinetic Curve Determination of the Combined Mutant Proteins 

To further study the effects of the combined mutation on the enzyme activity of PchEHA, a kinetic curve was created using (R)-SO and (S)-SO as the substrates (Table 3). The hydrolysis of epoxides catalyzed by these enzymes presented typical first-order reaction kinetics in agreement with the Michaelis–Menten equation. When (R)-SO was used as the substrate, the Michaelis constants (*K*_m_) of the combined mutants F1, F3, F4, F5, F7, F8, F10, and F13 were significantly lower than the *K*_m_ of the wt, indicating that the affinity of these eight combined mutants to the (R)-SO was higher than that of the WT. The *k*_cat_ values of F3, F4, F5, F7, F9, and F10 were higher than those of the wt, indicating that their catalytic efficiency was improved. In addition to F2, F6, F9, and F11, the *k*_cat_/*K*_m_ of all the other combined mutants increased, among which the *k*_cat_/*K*_m_ value of the combined mutants F3 and F5 was twice that of the wt, indicating that their conversion numbers and enzyme catalytic efficiencies were also much higher than those of the WT. 

When (S)-SO was used for the substrate, F1, F5, F12, and F13 exhibited lower *K*_m_ compared to that of the WT, indicating an enhanced substrate affinity for (S)-SO. Conversely, the combined mutant variants F4, F7, and F10 showed increased catalytic constants (*k*_cat_), due to enhanced catalytic efficiency. Ultimately, five mutants (F1, F7, F10, F12, and F13) displayed higher *k*_cat_/*K*_m_ values, significantly improving enzyme activity. The ratio of catalytic efficiencies (*k*_cat_/*K*_m_) for the hydrolysis of (R)/(S)-SO via epoxide hydrolase was referred to as the E-value, reflecting the enzyme’s substrate enantioselectivity parameter. Unlike the four combined mutant variants (F1, F9, F12, and F13) with slightly lower E-values compared to those of the wt, the E-values of the other nine mutant variants increased, indicating enhanced enantioselectivity for these nine combined mutants compared to that of the wt. Data indicated that the E-value of F3 was the highest; consequently, F3 may offer optimal enantioselectivity. Therefore, F3 was chosen for subsequent molecular docking studies.

### 2.7. Molecular Simulations Revealed the Mechanism of F3

**Figure 7:**
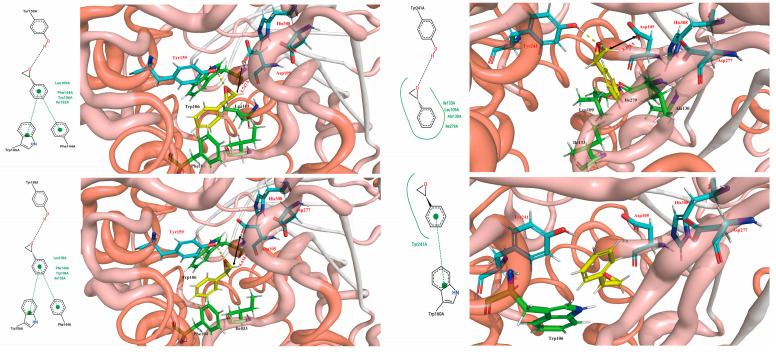
Molecular dynamics simulations carried out with the wt and F3 for (R)/(S)-SO. (**A**) 2D and 3D structural analysis of the interaction between the catalytic sites of the WT and (R)-SO; (**B**) 2D and 3D structural analysis of the interaction between the catalytic sites of F3 and (R)-SO; (**C**) 2D and 3D structural analysis of the interaction between the catalytic sites of the WT and (S)-SO; (**D**) 2D and 3D structural analysis of the interaction between the catalytic sites of F3 and (S)-SO.

The results of homology modeling and molecular docking indicated that PchEHA (Figure 1) could form hydrogen bonds between the hydroxyl group of Tyr159 and the epoxide ring of (R)/(S)-SO. Additionally, the carboxyl group of Asp105 nucleophilically attacked the carbon atom of the epoxide ring. A water molecule situated between Asp105 and His308 also nucleophilically attacked the carboxyl carbon in the transition state of Asp105, ultimately completing the catalytic reaction. However, in the F3 mutant, the Tyr residue was replaced from Tyr159 to Tyr241, which affected the conformational selection of (R)/(S)-SO. Based on the simulation results, it was found that (R)-SO could bind to the active cavities of both the wt and F3 enzymes, with binding free energies of −5.88 kcal/mol and −5.55 kcal/mol, respectively. For (S)-SO, the binding free energy in the active cavities of the wt and F3 enzymes was −5.65 kcal/mol and −5.35 kcal/mol, respectively. These results demonstrated that both wt and F3 were more inclined to catalyze the hydrolysis of (R)-SO. Further conformational analysis revealed that (R)-SO formed hydrogen bonds with Tyr159 in the wt and Tyr241 in F3, with a distance of approximately 3.7 Å to the carboxyl oxygen of Asp105. This distance facilitated the nucleophilic attack of the oxygen atom on the epoxide carbon, as shown in Figure 7A,B. Although (S)-SO could form a hydrogen bond with Tyr159 in the wt the distance to the carboxyl oxygen of Asp105 was 4.434 Å, which was not conducive to a catalytic reaction. Additionally, in F3, (S)-SO entered the active site in an inverted orientation, preventing a hydrogen bond from forming with Tyr241 and positioning itself far from the carboxyl oxygen of Asp105. This process may have inhibited the catalytic reaction. In conclusion, the Tyr241 residue in the mutated F3 enzyme was unfavorable for hydrogen bond formation with (S)-SO but enhanced conformational complementarity with (R)-SO, thereby increasing the enzyme activity and enantioselectivity of F3 toward (R)-SO.

## 3. Discussion

Compared to using traditional chemical catalysis for epoxides, biocatalysis with whole cells or enzymes offers high enantioselectivity and/or regioselectivity, with few or no byproducts [16]. EHs provide an environmentally friendly bioprocess for the preparation of (R)- or (S)-epoxides and ubiquitously exist in a variety of organisms, including common beans, potatoes, mouse livers, humans, fungi, and other species [17,18]. There are certain differences in the functions of EHs derived from different organisms or site localizations. EHs in eukaryotes are used to study the role of these enzymes in xenobiotic metabolism, signaling processes, and the production of optically pure drugs required by humans, while EHs in prokaryotes are necessary for catalytic pathways in which specific aromatic compounds or alkenes are used as carbon sources [19]. 

For the majority of wild-type EHs, achieving the catalytic activity and regioselectivity required for industrial applications is challenging work. Thus, improving the enzymatic activity for EHs has become a primary focus for researchers. The development of new techniques has improved the function of EHs, including the transition from directed evolution to a semi-rational or rational design. Tyr215 was replaced by Phe using site-directed mutagenesis, producing a Tyr215Phe mutant with improved enantioselectivity for EH from *Agrobacterium radiobacter* AD1 [20]. Based on a semi-rational design, the regiocomplementarity of GmEH3^W102V/P187F^ for (S)-1a was notably increased from 72.4% to 89.7% [21]. The white-rot basidiomycete fungus *P. chrysosporium* has been annotated with 20 genes encoding epoxide hydrolases. Among the 11 cloned epoxide hydrolases, PchEHA exhibited the highest enzymatic activity [22]. Semi-rational modification has been used to enhance the enzyme activity and enantioselectivity of PchEHA, but the results were not satisfactory [12]. Therefore, in this study, a mutant library for *PchEHA* was generated utilizing error-prone PCR and DNA shuffling techniques. A total of 38 strains with improved enzymatic activities or enantioselectivity were obtained. Analysis of the 38 mutants in the final screening revealed that the mutation sites were not limited to the vicinity of the active site, but were widely distributed in all parts of the entire PchEHA molecule. This result also verified the success of constructing a random mutation library. Interestingly, some of the mutation sites in the screened mutants were overlooked in previous studies, which may have differed in terms of their initial screening culture conditions or enzyme activity measurements.

Although the activities of some mutants against R-type substrates were improved, the activity against S-type substrates also increased for some mutants such as M31 and M32, leading to a decrease in enantioselectivity. The reasons for this result may be as follows: (1) the enantioselective changes were not changed in the first round of mutation [23]; (2) enantioselectivity is usually related to the superposition of certain amino acid residues around the active site [24], and those superimposed mutants were not found in the current libraries [25]; (3) the number of mutants able to be screened remains limited, and mutants with enantioselective changes were not found in this study. In addition, the enzyme activity analysis of various epoxides found that some mutants experienced a decrease in activity towards SO but better activity against other substrates of Ep (such as M13 and M15). This result is likely due to the changes at the mutation site affecting the access route of the SO substrate, thus providing an experimental basis for expanding the substrate range of PchEHA in the future.

A total of 13 combination mutants were obtained through merging multiple mutated amino acid sites via site-directed mutagenesis. Some multi-site mutants promoted the activity of PchEHA, but others weakened it [25]. The enzyme activity of the mutants M7, F5, and F7 on (S)-SO increased the enzyme activity by 0.8–1 times, indicating that the changes in the three sites of G14A, Y163H, and K291R may help to increase the activity of PchEHA. The enantioselectivity of the mutants M6, M21, M25, and F2 against SO increased greatly, showing that C108S and A189T may facilitate the enantioselectivity of PchEHA towards SO. The F3 mutant may have achieved the best E-value due to its three mutation sites. The protein homology modeling and molecular docking data of the F3 mutant also indicated that it may offer better catalytic activity and enantioselectivity for SO. The results reported in this study could be a useful starting point for future directed evolution experiments to generate novel EHs, and could provide insights into the source of selectivity for the future study of racemic epoxides.

## 4. Materials and Methods

### 4.1. Materials

rTaq DNA polymerase was purchased from Takara Biomedical Technology (Beijing, China) Co., Ltd. dITP, Dpn I, Tma endoV, and restriction enzymes were obtained from Thermo Fisher Scientific (San José, CA, USA), while 4-(4-Nitrobenzyl)pyridine 2×Phanta^®^ Max Master Mix Vazyme Biotech Co., Ltd. (Nanjing, China) and Racemic-SO, (R)/(S)-SO [26] were obtained from Sigma-Aldrich Co. (Saint Louis, MO, USA). (R)/(S)-Ep was obtained from Shenzhen Yawang Kangli Technology Co., Ltd. (Shenzhen, Guandong, China), and (R)/(S)-GT was obtained from Shanghai Kely Bio-Pharmaceutical Co., Ltd. (Shanghai, China). The *Escherichia coli* strain JM109 served as the host for cloning. *Escherichia coli* BL21 (DE3) (Novagen, Gibbstown, NJ, USA) was used for gene expression. The primers used for mutagenesis PCR in this study were synthesized at TSINGKE (TSINGKE Biological Technology, Chengdu, China). The nucleotide sequences of primers used to amplify the *PchEHA* gene were EHAF (5′-GCAAATGGGTCGGATCCATGG-3′; restriction site, BamH I) and EHAR (5′-CGTGTGCGGCCGCAAGCTTCTAC-3′; restriction site, Hind III).

### 4.2. Mutation Library Construction 

The expression plasmid pE28EHA [pET-28a (+), hosting the gene encoding *PchEHA*] was used as the template for cloning the mutation genes [22]. In addition, dITP as a mutagenic agent and dNTP/dITP were included in ratios of 4:0, 3:5, 1:3, and 1:7, respectively [23]. Mutagenesis PCR was performed with rTaq DNA polymerase using a program of 4 min at 94 °C followed by 30 cycles of 94 °C for 30 s, 54 °C for 30 s, and 72 °C for 60 s, and a final extension at 72 °C for 10 min. Tma endo V was added into the products to cut mutated DNA into fragments after Dpn I was used to remove the non-mutated template. Assembly of the digested DNA fragments was carried out via PCR reaction without any primers, followed by 45 cycles of 94 °C for 30 s, 42 °C for 30 s, and 72 °C for 30 s (plus 2 s per cycle), and a final extension at 72 °C for 10 min. The assembly products were directly used as a template for the subsequent amplification reaction with the primers (EHAF and EHAR), followed by 25 cycles of 94 °C for 30 s, 52 °C for 30 s, and 72 °C for 90 s, and a final extension at 72 °C for 10 min. The reamplified PCR products and pET-28a(+) were digested with BamH I and Hind III, then quickly ligated with T4 DNA ligase. Next, the ligation mixture was transformed into competent cells of *E. coli* DH5α. All colonies were saved as a mutant library of the *PchEHA* gene for subsequent experiments. A total of 34 colonies of *E. coli* DH5α were randomly selected for sequencing to explore the mutagenic efficiency of *PchEHA* in the mutant library.

### 4.3. Mutation Library Screening

The recombinant plasmid pE28EHA was extracted and transformed into *E. coli* BL21 (DE3), and protein expression was induced at a final concentration of 0.5 mM isopropyl-β-D-thiogalactopyranoside (IPTG) at 18 °C. The enzyme activity of whole cells was determined in the preliminary screening [27]. The fermentation liquid (1 mL) was centrifuged at 4 °C, and collected cells were suspended in 200 µL of buffer (0.1 M K_2_HPO_4_/KH_2_PO_4_, pH 8.0). A 200 µL reaction solution, defined as 100 µL of the cell suspension, 3 µL of 800 mM (R)- SO or (S)-SO, and 97 µL of the buffer, was left to react at 37 °C for 30 min. After centrifugation, 50 μL of the supernatant, 145 μL of the buffer, and 5 μL of 200 mM sodium periodate were mixed well, and the OD value was measured at 290 nm [28]. The ratio of the hydrolysis rate of (R)-SO or (S)-SO was considered approximately equivalent to the enantioselectivity of the enzyme.

The same volume of cells was collected for ultrasonic lysis as in the preliminary screening (with a 5 s pause after each 3 s ultrasonic pulse, for a total processing time of 10 min at 35% power) for re-screening. The lysed liquid was centrifuged at 4 °C (10,000 rpm for 10 min) once the cells were completely lysed. Then, enzyme activity was determined according to the above method. After two rounds of screening, the mutants with significantly improved enzyme activity (>20%) were inoculated in 50 mL LB medium and cultured at 37 °C by shaking until the OD600 was 0.6. IPTG was then added to induce protein expression (18 °C at 200 rpm for 24 h). The cells were lysed via ultrasound under an ice bath, and the lysed liquid was centrifuged at 1000 rpm for 10 min. Then, the enzyme activity was measured according to the aforementioned enzyme activity measurement method, enabling the enzyme-activity-enhancing strains to be tested once more. 

### 4.4. Protein Expression and Purification

Protein induction and expression procedures followed the pET system operation manual [28]. Expression was induced at a final concentration of 0.5 mM IPTG at 18 °C for 24 h. Protein purification was conducted using the ÄKTAPrime system (GE Healthcare, Chicago, IL, USA) and a HisTrap FF Crude 1 mL column with immobilized nickel ions. Recombinant proteins were eluted with 0–1 M imidazole. The eluates with an absorption peak at 280 nm were combined and dialyzed overnight at 4 °C with 2 µL of dialysis buffer [10 mM K_2_HPO_4_/KH_2_PO_4_, pH 8.0, 250 mM NaCl, 10% glycerol, and 0.1 mM ethylenediamine tetra-acetic acid (EDTA)]. The protein sample was concentrated to an approximately 1 mL final volume with polyethylene glycol 20,000. The protein was quantified using a Bradford protein content detection kit, and then the protein samples from each mutant were adjusted to the same concentration.

### 4.5. Enzymatic Determination

The enzyme activity of PchEHA was determined using the 4-p-nitrobenzylpyridine colorimetric method [29]. The 50 µL total reaction volume consisted of 45 µL buffer, 2 µL substrate (800 mM SO, 300 mM GT, 300 mM Ep), and 10 µg enzyme. The reaction was carried out in a 37 °C water bath for 30 min. Then, the mixture was immediately cooled, and 50 μL of enzyme activity reagent A (50 mM 4-p-nitrobenzylpyridine, 80% ethylene glycol, 20% acetone) was added. The mixture was placed into an 80 °C water bath for 10 min and cooled with running water. Next, 60 µL of the reaction solution was added to 840 L of the buffer and 300 L of enzyme activity reagent B (50% triethylamine, 50% acetone), followed by a reading of the OD565. Each reaction was performed at least three times, and the enzyme activity was calculated according to a standard curve.

### 4.6. Site-Directed Mutagenesis

The specific primers (see Supplementary Data, Table S1) were designed according to the sequence of mutants after testing enzyme activity, and the pE28EHA plasmids containing the corresponding mutation sites were used as templates for PCR amplification to obtain site-directed mutant genes. The PCR reaction was performed with 2×Phanta^®^ Max Master Mix using a program of 30 s at 95 °C followed by 30 cycles of 95 °C for 15 s, 55 °C for 15 s, and 72 °C for 60 s, with a final extension at 72 °C for 5 min.

### 4.7. Determination of Kinetic Properties

The expression, purification, and enzyme activity of recombinant mutant proteins were assessed according to the above method. Next, 20 µL of the reaction solution (2.5 g recombinant mutant protein, 10 to 80 mM SO) was incubated at 37 °C for 0, 2, 4, 6, and 8 min, followed by the addition of 20 µL enzyme activity reagent A. The mixture was left to react in a water bath at 80 °C for 10 min and then immediately placed in ice water for cooling. Then, 30 μL of the above reaction solution was added to 470 μL of 0.1 M potassium phosphate buffer (pH 8.0), followed by adding 150 µL of enzyme activity reagent B. Before measuring the OD value at 565 nm with a spectrophotometer, 250 µL of the mixed solution was added into the microplate. The enzymatic activities were calculated according to the standard curve produced using the same procedure above, with each substrate, in a varied range of concentrations. The initial reaction rate was calculated using the resultants produced at different reaction times. Then, the *V*_max_ and *K*_m_ values were calculated using the GrahPadPrism5.0 software. All parameters were calculated using the mean values from three independent experiments.

### 4.8. Structure Analysis and Molecular Docking 

The three-dimensional structures of PchEHA and combined mutant F3, constructed with Modeller 10.1, were retrieved from the PDB database (PDB ID: 4JNC, 4C4X, 3ANS, 3PDC, 6I5E), and the optimized model protein structure was evaluated using the PROCHECK program. The 3D coordinates of (R)/(S)-SO for subsequent docking were generated using Auto-DockTools 1.5.6. The molecular docking of wt epoxide hydrolase and F3 epoxide hydrolase with (R)/(S)-SO were performed by AutoDock 4.2.6. The substrate conformation in the complex structures was based on previous reports [25].

## Figures and Tables

**Table 1:** Statistics of *PchEHA* mutation types.

Mutant Type	Base Changes	Number	Percentage
Transition	A-G	48	27.91
	G-A	33	19.19
	C-T	39	22.67
	T-C	32	18.60
Transversion	T-A	2	1.16
	C-A	3	1.74
	C-G	6	3.48
	A-T	3	1.74
	G-T	3	1.74
	G-C	3	1.74

**Table 2:** Identification of the mutation sites for PchEHA.

Mutant	Mutant Sites	Mutant	Mutant Sites
M1	A189T	M20	G167D/T245A
M2	G82C/S116G/A330T	M21	A60T/A189T/T198M/A287T
M3	D124G	M22	N16D/K192R/V318I
M4	A3V/H93Y	M23	I139V/Q151R/E323G
M5	G153R	M24	R13H/I136V/P188S/A330V
M6	L145P/A189T	M25	K161R/A273T/D305G
M7	Y163H	M26	A130V/Q150R/L284S
M8	Y117C/R242K	M27	G26A/A306T
M9	P119H	M28	G14A/K291R
M10	I299V	M29	E220G/A283V
M11	S131P	M30	A24T
M12	L31F/K192M	M31	I123T/K149R/D305G
M13	P138L/F221S	M32	K98E/S164G/K223E
M14	A3V/N230S	M33	G103D/D316G
M15	G82S/W209R	M34	A76P/S80L/A126V/S131P/E165G/A273T/D316E
M16	D74G/E293V	M35	F15S/G26D/A130T/G167S/M244I/F329L
M17	L176S	M36	G107D/A129T/D187G/K192M/L206P/F270L/D292G/W294R
M18	A330S	M37	S53G/S110L/G134D/I148V/T218S/A250V/I256V/G332E
M19	E327G	M38	D2G

**Table 3:** Kinetic parameters of PchEHA and 13 combined mutants catalyzing the hydrolysis of SO enantiomers.

	(R)-SO			(S)-SO			E-Value
Mutant	*K*_m_ (mM)	*K*_cat_ (s^−1^)	*k*_cat_/*K*_m_ (s^−1^·mM^−1^)	*K*_m_ (mM)	*k*_cat_ (s^−1^)	*k*_cat_/*K*_m_ (s^−1^·mM^−1^)	-
wt	25.75 ± 2.08	1738.40 ± 50.79	67.51	39.23 ± 4.49	1351.63 ± 68.47	34.45	1.96
F1 (G82C/C108S/S116G/A330T)	21.9 ± 2.71	1775.98 ± 73.05	81.10	27.38 ± 3.05	1349.58 ± 56.08	49.29	1.65
F2 (C108S/K161R/A273T/D305G)	30.36 ± 0.95	1506.07 ± 18.46	49.61	68.35 ± 8.39	1253.92 ± 86.03	18.35	2.70
F3 (G14A/C108S/K291R)	21.71 ± 2.6	3023.75 ± 119.99	139.28	51.04 ± 2.04	1003.82 ± 19.95	19.67	7.08
F4 (G82C/S116G/Y163H/A330T)	20 ± 4.44	2101.25 ± 148.08	105.06	77.82 ± 11.99	1910.60 ± 172.81	24.55	4.28
F5 (Y163H/E251G)	14.16 ± 1.19	2349.98 ± 52.27	165.96	30.39 ± 2.99	986.05 ± 37.98	32.45	5.11
F6 (K161R/Y163H/A273T/D305G)	38.24 ± 3.51	1263.48 ± 50.66	33.04	93.45 ± 12.21	1336.60 ± 198.71	14.30	2.31
F7 (G14A/Y163H/K291R)	20.36 ± 1.34	2483.92 ± 52.43	122.00	57.82 ± 7.75	2080.07 ± 145.89	35.97	3.39
F8 (G82C/S116G/E251G/A330T)	16.28 ± 2.45	1697.40 ± 72.77	104.26	41.4 ± 3.53	1409.03 ± 54.45	34.03	3.06
F9 (K161R/E251G/A273T/D305G)	76.34 ± 9.38	1819.03 ± 130.31	23.83	52.71 ± 7.07	1195.83 ± 80.84	22.69	1.05
F10 (G14A/E251G/K291R)	22.6 ± 1.75	2250.22 ± 59.03	99.57	41.96 ± 2.07	1605.83 ± 36.09	38.27	2.60
F11 (A24T/E251G)	26.28 ± 3.79	1657.77 ± 87.26	63.08	43.98 ± 3.94	1137.75 ± 47.52	25.87	2.44
F12 (I123T/K149R/E251G/D305G)	22.45 ± 0.88	1777.35 ± 23.40	79.17	21.98 ± 1.01	1093.33 ± 16.68	49.74	1.59
F13 (K98E/S164G/K223E/E251G)	20.92 ± 2.08	1622.92 ± 52.49	77.58	28.65 ± 2.65	1390.58 ± 49.01	48.54	1.60

## Data Availability

The data are included in the figures and tables of this manuscript.

## Supplementary Materials

The following supporting information can be downloaded at: https://www.mdpi.com/article/10.3390/molecules29204864/s1, Table S1: The oligonucleotide primers used for error PCR and site-directed mutagenesis.
