# Supplementary material for: Improving Hydrolytic Activity and Enantioselectivity of Epoxide Hydrolase from Phanerochaete chrysosporium by Directed Evolution
Source: Molecules. 2024 Oct 14;29(20):4864. doi: 10.3390/molecules29204864 (PMC11509930; doi:10.3390/molecules29204864)
Supplement: Supplementary file 1 [file molecules-29-04864-s001.zip › molecules-3134717-supplementary.pdf]

Supplementary Table S1

Table S1. The oligonucleotide primers used for error PCR and site-directed mutagenesis

Primer name	Oligo nucleotides (5′-3′)	Note
EHA-F	5′-GCAAATGGGTCGGATCCATGG-3′	The forward primer used for error-prone PCR, and the underlined sequence are the restrictive cleavage site *BamH* I
EHA-R	5′-CGTGTGCGGCCGCAAGCTTCTAC-3′	The reverse primer used for error-prone PCR, and the underlined sequence are the restrictive cleavage site *Hind* III
C108S-F	5′-ACTGGGGCAGCTTGTCGGTCTCGCGAC-3′	The forward primer used for site-directed mutagenesis of C108S
C180S-R	5′-ACCGACAAGCTGCCCCAGTCATGGCCGAT-3′	The reverse primer used for site-directed mutagenesis of C108S
Y163H-F	5′-GGAAGTTCCACAGCGAGCCTGGCCTG-3′	The forward primer used for site-directed mutagenesis of Y163H
Y163H-R	5′-GGCTCGCTGTGGAACTTCCAGTAGCCGTACA-3′	The reverse primer used for site-directed mutagenesis of Y163H
E251G-F	5′-TGCAGGCGGGCGACGACAAACAAATCCTACC-3′	The forward primer used for site-directed mutagenesis of Y163H
E251G-R	5′-TTGTCGTCGCCCGCCTGCAGCCCGCTC-3′	The reverse primer used for site-directed mutagenesis of Y163H
